# Clinicopathological value of the upregulation of cyclin-dependent kinases regulatory subunit 2 in osteosarcoma

**DOI:** 10.1186/s12920-022-01234-8

**Published:** 2022-04-11

**Authors:** Chaohua Mo, Yanxing Wu, Jie Ma, Le Xie, Yingxin Huang, Yuanyuan Xu, Huizhi Peng, Zengwei Chen, Min Zeng, Rongjun Mao

**Affiliations:** 1 Department of Pathology, Foshan Hospital of Traditional Chinese Medicine, Guangzhou University of Chinese Medicine, Foshan, 528300 Guangdong China; 2 Department of Medical Oncology, The First Affiliated Hospital of Guangxi Medical University, Nanning, Guangxi Zhuang Autonomous Region China

## Abstract

**Background:**

Cyclin-dependent kinase subunit 2 (CKS2) is a member of cyclin dependent kinase subfamily and the relationship between CKS2 and osteosarcoma (OS) remains to be further analyzed.

**Methods:**

80 OS and 41 non-tumor tissue samples were arranged to perform immunohistochemistry (IHC) to evaluate CKS2 expression between OS and non-tumor samples. The standard mean deviation (SMD) was calculated based on in-house IHC and tissue microarrays, and exterior high-throughput datasets for further verification of CKS2 expression trend in OS. The effect of CKS2 expression on clinicopathological parameters of OS patients, and single-cell in OS tissues was analyzed through public high-throughput datasets and functional enrichment analysis was conducted for co-expression genes of CKS2 in accordance with weighted correlation network analysis.

**Results:**

A total of 217 OS samples and 87 non-tumor samples (including tissue and cell line) were obtained from in-house IHC, microarrays and exterior high-throughput datasets. The analysis of integrated expression status demonstrated up-regulation of CKS2 in OS (SMD = 1.57, 95%CI [0.27–2.86]) and the significant power of CKS2 expression in distinguishing OS samples from non-tumor samples (AUC = 0.97 95%CI [0.95–0.98]). Clinicopathological analysis of GSE21257 indicated that OS patients with higher CKS2 expression was more likely to suffer OS metastasis. Although Kaplan–Meier curves showed no remarkable difference of overall survival rate between OS patients with high and low-CKS2, CKS2 was found up-regulated in proliferating osteosarcoma cells. Co-expression genes of CKS2 were mainly assembled in function and pathways such as cell cycle, cell adhesion, and intercellular material transport.

**Conclusions:**

In summary, up-regulation of CKS2 expression in OS tissue was found through multiple technical approaches. In addition, scRNA-seq and co-expression analysis showed that CKS2 may have an impact on important biological process linked with cell cycle, cell adhesion, and intercellular material transport. Present study on CKS2 in OS indicated a promising prospect for CKS2 as a biomarker for OS.

**Supplementary Information:**

The online version contains supplementary material available at 10.1186/s12920-022-01234-8.

## Introduction

Osteosarcoma (OS) is the most common primary malignant bone tumor that occurs in children and adolescents. The annual incidence rate in the world is about 1–3 per million [1]. The pathogenesis of OS remains unclear, while overgrowth of bone, chromosomal abnormalities, mutation of tumor suppressor gene, transcription factors, and miRNAs play an important role in the development of osteosarcoma [2]. OS is highly malignant, progresses rapidly after the onset and it has a poor prognosis accompanied by a tendency of lung metastasis [3]. With the progress of surgery, chemotherapy, and radiotherapy, the long-term survival rate of OS patients has been greatly improved [4]. However, the overall prognosis of OS patients is still poor, especially those with distant metastasis or recurrent cancer [5]. Therefore, identifying the molecular mechanism of OS advances the development of new diagnostic methods and treatment strategies.

Cyclin-dependent kinases (CDK) is a serine/ threonine protein kinase which is involved in the process of cell cycle [6]. CDK regulates the cell cycle transition and makes the cell cycle proceed orderly [7]. In particular, CDK4/ 6 plays an important role in OS cell proliferation by controlling the transition from G1 phase restriction point to S phase [8]. The occurrence and development of OS is the result of multiple factors where amplification of CDK4 has been demonstrated exerting chromosome variation, methylation, gene mutation and cytokine up-regulation [9, 10]. Cyclin-dependent kinases regulatory subunit 2 (CKS2) is a protein that interacts with cyclin-dependent kinases and is essential for cell cycle regulation [11]. CKS2 can positively regulate cell proliferation, invasion, and migration to promote the progression of certain cancers [12]. CKS2 is highly expressed in a variety of malignant tumors, including hepatocellular carcinoma [13], esophageal cancer [13, 14], gastric cancer [15], colorectal cancer [16–18] and bladder cancer [19, 20].Various studies have shown that the upregulation of CKS2 is closely related to tumor size, histological grade, and pathological tumor-node-metastasis (pTNM) staging [12].

At present, there were no reports on the relationship between CKS2 and OS, only Pan Y et al. spotted that CKS2 could be a key regulatory factor in OS based on protein–protein interaction (PPI) analysis, while the conclusion is not reliable owing to the lack of experimental evidence. For this reason, the clinical significance and molecular mechanism of CKS2 requires a comprehensive investigation in OS. In current study, protein level of CKS2 in OS clinical samples was detected from our institute with immunohistochemistry (IHC). Secondly, the protein level of CKS2 was compared to its mRNA level using public high-throughput data. Lastly, single-cell (SC) RNA-seq analysis and prospective mechanism of CKS2 in OS was explored with in-silico mining.

## Patients and methods

### Verification of CKS2 protein expression in OS tissues

The samples tested by IHC experiments were all collected from the wax blocks (80 cases) surgically-resected OS specimens derived from the Department of Pathology, Foshan Hospital of Traditional Chinese Medicine, Guangdong Province between January 2008, and May 2021. There were 50 cases of osteocytes OS tissue, 11 cases of chondrogenic OS tissue, 9 cases of fibroblastic OS tissue, and 10 cases of other types (Related file 1). The control groups were benign osteochondroma samples for osteochondroma is an ideal control for OS including cartilage tissue, bone tissue and benign mesenchymal tissue, which were excised with the consent of patients. These specimens all met the following conditions:

1. The patient was finally diagnosed with OS by a series of pathology detection.
2. Complete clinical data could be acquired from the OS specimen.
3. The OS patients didn’t receive chemotherapy or radiotherapy before surgery.

The procedure of immunohistochemical (IHC) staining was as follows. Firstly, paraffin sections were dewaxed with xylene, absolute ethanol, and alcohol (70–95%), and then soaked with distilled water. EDTA antigen repair buffer was used to repair the antigen, and attention should be paid to prevent excessive evaporation of the buffer. After natural cooling to room temperature, the slices were washed with PBS buffer. Then the slices were incubated in 3% dioxygen water at room temperature to block the endogenous peroxidase. After that, the tissue was evenly covered with 3% BSA and sealed at room temperature for 30 min, and CKS2 antibody (rabbit polyclonal antibody, 1:500 dilution) prepared with PBS was dripped onto the slices. Next, the slices were placed in a wet box and incubated overnight at 4 ℃. After incubation at room temperature for 50 min, fresh DAB chromogenic solution was added. After the color development confirmed via microscope observation, the sections were washed to stop color development. Afterwards, the slice was retained with Harris hematoxylin for 3 min and differentiated by 1% hydrochloric acid alcohol for several seconds. Finally, alcohol (70–95%), anhydrous ethanol and xylene were used in turn to dehydrate the slices, then the slices were dried and sealed with neutral gum.

Under the magnification of 200 times, the IHC sections of each patient were randomly evaluated for 5–10 fields with dense and non-repetitive cells. At least 100 cells were counted, and the percentage of positive cells was recorded. The score was scored according to the following criteria: 1—proportion of positive cells in the total cells was less than 25%; 2—positive cells accounted for 25–50% of the total cells; 3—positive cells accounted for 50–75% of the total cells; 4—positive cells accounted for more than 75% of the total cells. According to the staining intensity of positive tumor cells in each section, the score was 0—cells were not stained; 1—cells were stained light yellow; 2—cells were stained brown yellow; 3—cells were stained brown. The scoring work was completed by two pathologists while positive cell count and staining intensity were evaluated respectively. If there was difference in the scores of the two pathologists, they would count again until they got the same result. The final score of each OS section was obtained by multiplying the positive cell percentage score and staining intensity score.

### Verification of CKS2 mRNA expression in OS

3 pairs of tumor and non-tumor specimen was collected from OS patients admitted to the Department of Orthopedic Surgery of The First Affiliated Hospital of Guangxi Medical University from 10 October 2015 to 18 July 2017. The tumors and non-tumor tissues were surgically excised with the consent of patients. The comparative analysis of mRNA expression in these 6 cases was conducted with microarray technology provided by Shanghai Kangcheng Biological Company.

The search was carried out for datasets of mRNA microarrays or high-throughput sequencing for human OS published before 30 June 2021 in databases including Genomic Data Commons (GDC, https://portal.gdc.cancer.gov/), Gene Expression Omnibus (GEO, https://www.ncbi.nlm.nih.gov/geo/), ArrayExpress (https://www.ebi.ac.uk/arrayexpress/), and Oncomine (https://www.oncomine.org/). The expression analysis of CKS2 mRNA incorporated datasets contained more than three tissue samples or cell line samples of OS, and included the data of non-tumor tissues or cells in the control group. Simultaneously, the corresponding clinical parameters of OS patients in the dataset were extracted. The elimination of batch effects in the same platform was carried out with ComBat function in sva of R package [21]. The expression trends between cancer and control group were displayed using box plots.

Random effects model from STATA 14.0 (StataCorp,Texas,USA) was used to calculate the standardized mean difference (SMD) and 95% confidence interval of CKS2 expression between OS and non-tumor specimens in all included datasets. Effect of SMD and standard error of SMD effect were inputted as variables to draw funnel chart judging whether the datasets included were biased.

SPSS 25.0(IBM SPSS, Armonk, NY, USA) was employed to draw the receiver operator characteristic (ROC) curve, reflecting the sensitivity and specificity of CKS2 mRNA expression based on different thresholds. Midas command of STATA 14.0 was applied to draw forest plots to reflect the sensitivity and specificity of each dataset. At the same time, the fourfold table data of the diagnostic test of each dataset were integrated to draw summarized receiver operator characteristic (sROC) curve to comprehensively reflect the ability of CKS2 to distinguish OS tissues from non-tumor tissues.

Finally, integrative analysis was conducted on CKS2 mRNA expression and IHC results in all datasets to determine whether the expression of CKS2 mRNA was consistent with that on protein levels.

### Analysis of the relationship between the expression of CKS2 mRNA and the clinical characteristics as well as the survival of OS patients

The Htseq-Counts data of the TARGET-OS project were downloaded from the GDC management page (https://portal.gdc.cancer.gov/GDC), and of R package dplyr (https://dplyr.tidyverse.org/) was used to convert the raw counts data into transcripts per kilobase of exon model per million mapped reads (TPM) formation. In addition, datasets containing the pathological grade, metastasis, and survival of OS patients from GDC, GEO, Array Express databases, and Oncomine databases were searched to analyzed the clinical significance of CKS2 mRNA expression. Besides, R package Survival (https://www.rdocumentation.org/packages/survival/versions/2.42-3) was adopted to conduct Kaplan–Meier (KM) analysis for patients divided into two groups according to the median of CKS2 expression, and the survival curves of the conditional probability were drawn. SMD and integrated hazard ratio (HR) were arranged to reflect the relationship between CKS2 mRNA and metastasis as well as survival. *P* < 0.05 was considered statistically significant.

### Exploring CKS2 expression in OS cells by scRNA-seq analysis

The GSE152048 dataset of GEO contains OS tissues for dissecting the cells’ transcriptional traits. The subpopulations of cells were obtained through linear dimensionality reduction principal component analysis (PCA) and uniform manifold approximation and projection (UMAP) clustering. The marker genes and disease difference molecules in the cell subpopulations were analyzed and screened using the Wilcox rank-sum test. The marker genes of each population obtained in this dataset were compared with the marker molecules collected in the paper providing raw data and CellMarker (http://biocc.hrbmu.edu.cn/CellMarker/index.jsp). According to the relative expression of the single-cell genes after integrating and correcting the batch effect, the differentially expressed gene set was selected as the variable to conduct the trajectory construction function and construct the single-cell development trajectory diagram in Monocle 2 software package, which was displayed with 2D visual results.

### Immune infiltration analysis reflecting the role of CKS2 in tumor microenvironment (TME) of OS

The TIMER2.0 database [22] (http://timer.cistrome.org/), a tool could evaluate the immune infiltration of various cancer types comprehensively, offering a variety of methods to evaluate the degree of immune infiltration, which could help researchers systematically explore the immunomics and genomics features of tumors. This website preferred the expression matrix in the original TPM formation as the input file. Therefore, the expression matrix of TARGET-OS in TPM formation was uploaded into the website, and TIMER, xCell, Microenvironment Cell Populations-counter, CIBERSORT, EPIC and quanTIseq were applied to predict the immune cell component. By calculating the Pearson correlation coefficient, components highly correlated with CKS2 mRNA expression in the TME were determined to explore the impact of CKS2 on TME.

### Exploration of the biological pathways and functions of CKS2 co-expression genes based on weighted correlation network analysis (WGCNA)

WGCNA [23] was utilized to analyze the expression correlation coefficients between CKS2 mRNA and other parts in expression matrix for investigating potential co-expression genes that may participate in the same biological process. The analysis steps were as follows: (1) Calculate the correlation coefficient between all genes in expression matrix (2) Transform the expression matrix into adjacency matrix by power exponential weighting analysis (3) Transform the adjacency matrix into topological matrix (4) Use node dissimilarity to perform cluster analysis and identify network modules, to discover the potential co-expression genes of CKS2 in OS. At the same time, module eigengene (ME), as principal component of module network, represented the expression trend of entire module. Module membership (MM) represented the correlation between the expression profile of a single gene and ME, and the gene with high level MM was classified as the key gene.

Through gene ontology (GO) database, the biological functions of co-expressed genes were summarized from perspectives of molecular function (MF), biological process (BP), and cell composition (CC). Meanwhile, the goSim function of in R package GoSemSim [24] was used to calculate the semantic similarity between each enriched GO terms, and GO terms with high similarity were clustering.

R package Clusterprofiler [25] was employed to perform enrichment analysis in the Kyoto Encyclopedia of Genes and Genomes (KEGG) biological pathway of co-expressed genes, and the latest version of annotation information for three-level signal pathways on the KEGG official website would help sort out the enrichment pathways, and R package ggplot2 was applied to visualize the results.

### Statistical analysis

In this study, in addition to the statistical analysis explained above, when the CKS2 expression presented a normal distribution, R 4.0.3 was used for Student's t test. If CKS2 expression was not normally distributed, a non-parametric test was used to compare the differential expression of CKS2 in OS and non-tumor tissues. GraphPad Prism 8.0 (GraphPad Software, San Diego, CA, USA) was employed to draw violin chart showing the distribution of CKS2 expression in OS patients with different clinical characteristics, and the distribution differences of components of tumor immune infiltration in different CKS2 expression groups. SPSS 25.0 was used to draw ROC curves, reflecting the sensitivity and specificity of CKS2 mRNA expression value in the corresponding model under different threshold states. Area under curve (AUC) was used to evaluate the ability of CKS2 expression to distinguish between OS and non-tumor tissues. STATA 14.0 was employed to calculate SMD and 95% confidence interval, systematically reflecting the expression of CKS2 and sROC was calculated to illustrate the ability of CKS2 to distinguish OS from non-tumor tissues. Log-rank test was utilized to determine whether these survival curves could reveal the difference in prognosis of patients of different groups. Fisher’s exact test was conducted to test the results of biological function and pathway enrichment analysis. *P* < 0.05 was regarded as the criterion to determine whether the analysis result was statistically significant.

## Results

### IHC verified the up-regulated expression of CKS2 protein in OS tissues

**Fig. 1:**
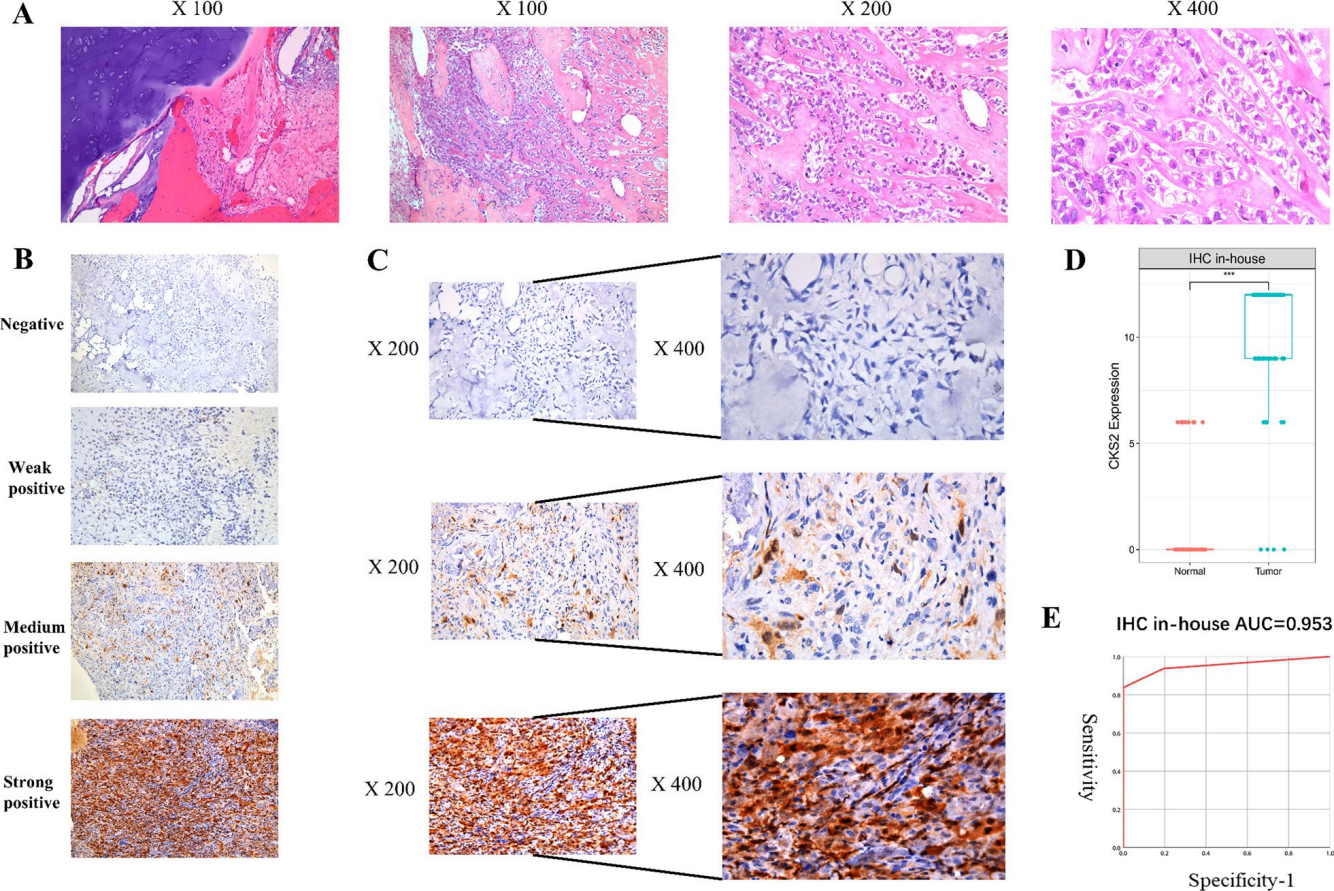
Hematoxylin–eosin (HE) staining and Immunohistochemistry (IHC) staining of CKS2 in osteosarcoma (OS) and non-tumor tissues. **A** HE staining of CKS2 in non-tumor tissues (100×) and OS tissues (100×, 200×, 400×). **B** Plots showing the staining intensity prospectively scored by pathologists. **C** Negative IHC staining of CKS2 in non-tumor tissues (200×,400×); Moderate positive IHC staining of CKS2 in OS tissues (200×,400×); Strong positive IHC staining of CKS2 in OS tissues (200×,400×). **D** Box-plot of IHC staining displayed expression analysis of CKS2. **E** The receiver operator characteristic (ROC) curve of IHC staining displayed expression analysis of CKS2

Among 80 specimens of OS, 93.75% showed moderately or strongly positive immunostaining of CKS2, while in non-tumor samples, 80.49% were stained negatively or weakly positive, and no strongly positive tissues were observed (Fig. 1A–C). By comparison between IHC of CKS2 expression in 80 OS and 41 non-tumor tissues, it was found that the expression of CKS2 in OS tissues was significantly higher than that in non-tumor tissues (Fig. 1D, *P* < 0.005). The high expression of CKS2 could distinguish OS from non-tumor tissues fairly (Fig. 1E, AUC = 0.953).

### Up-regulated expression of CKS2 mRNA in OS verified by microarray and public datasets

**Fig. 2:**
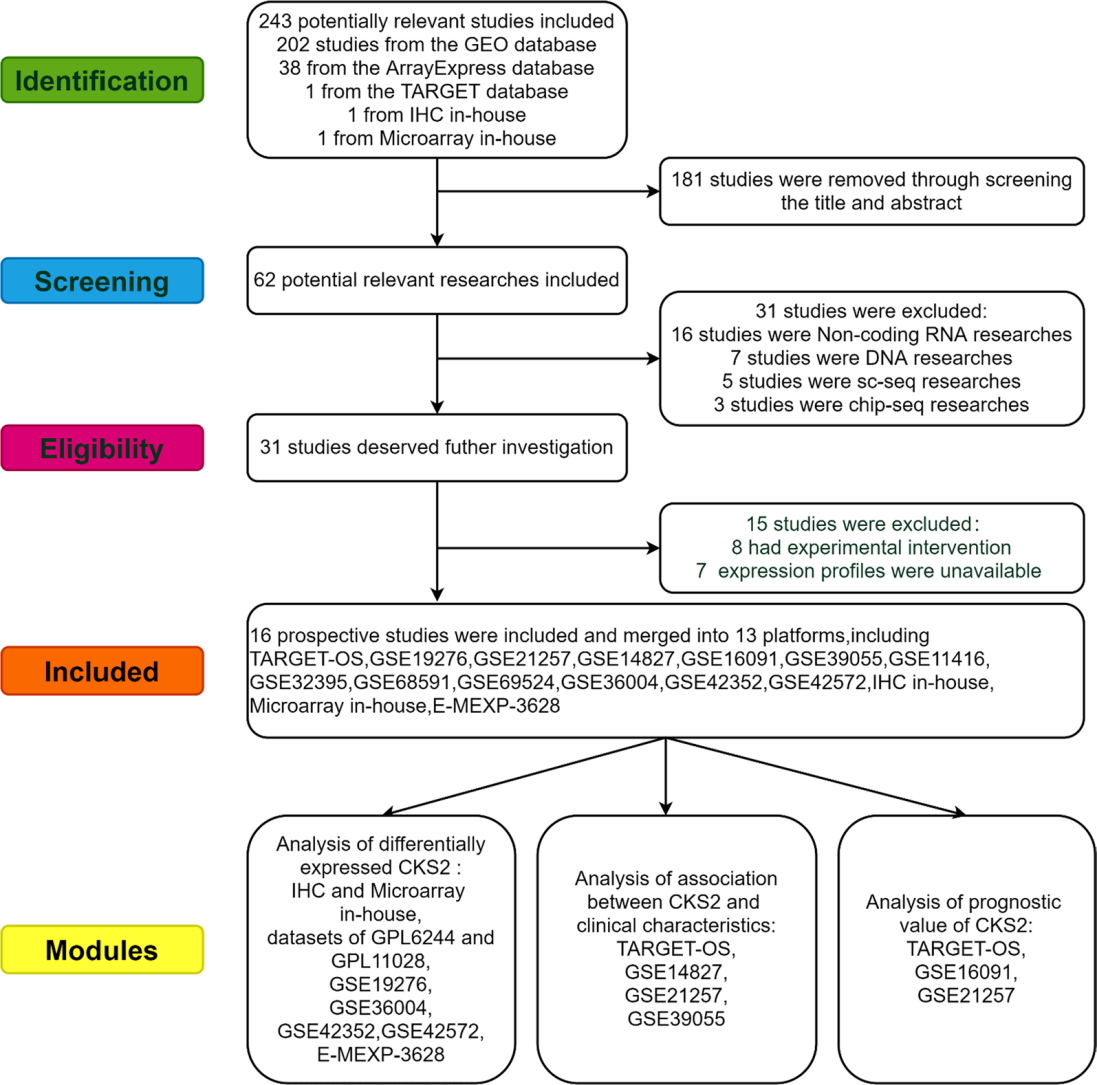
Flow chart of data collection for this study

**Fig. 3:**
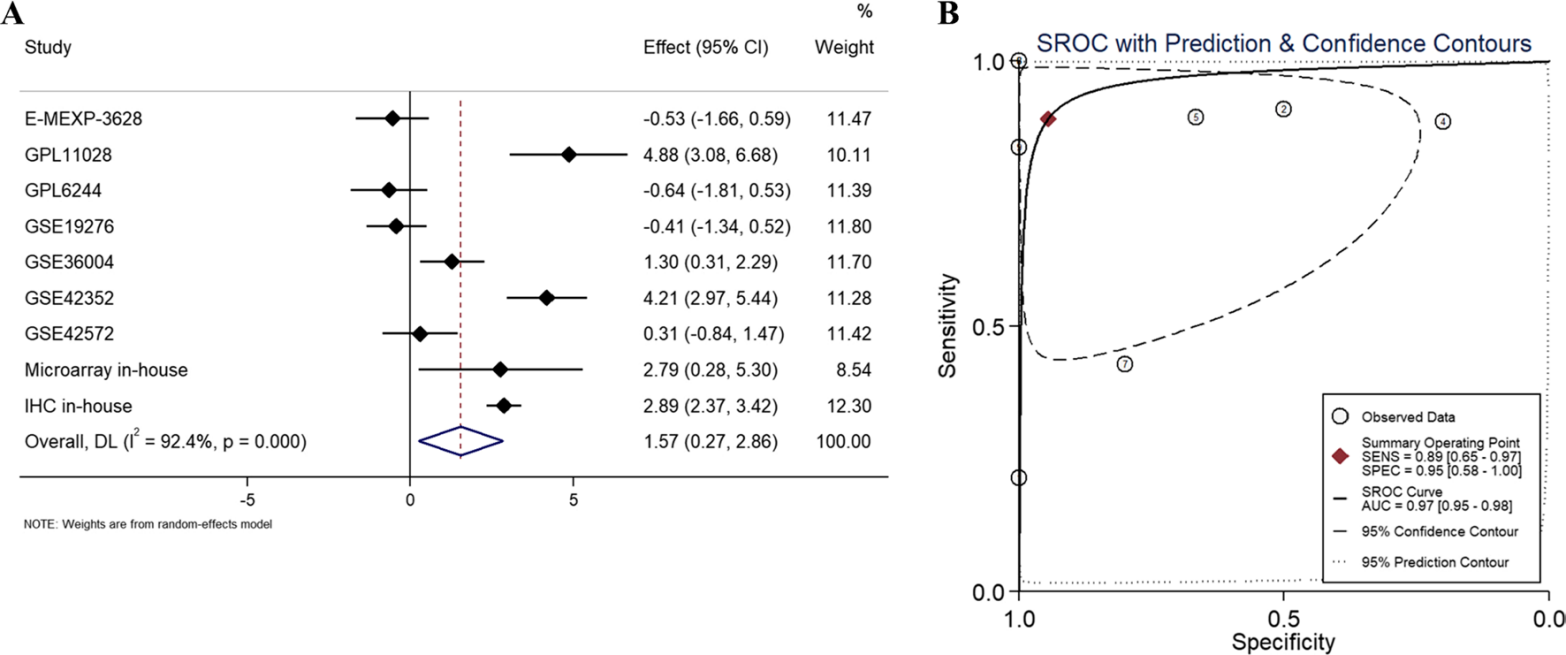
Pooled standard mean deviation (SMD) forest plot and summary receiver operating characteristic (sROC) curves of CKS2 in all types of OS for in-house tissue microarray, external microarrays and IHC staining. **A** Pooled SMD forest plot reflected overexpression of CKS2 in OS. **B** sROC curve reflected discriminatory ability of CKS2 expression in distinguishing osteosarcoma from non-tumor tissue

Analysis of microarray in-house showed that expression of CKS2 mRNA in OS tissues had an elevated trend (Additional file 1: Fig. S1), and the ROC curve (AUC = 1, Additional file 1: Fig. S2G) indicated that it possessed a strong ability to distinguish OS from non-tumor tissues. In the public database, a total of 9 mRNA microarrays, including 134 OS samples and 39 non-cancer samples (Table 1) were used to obtain expression trend of CKS2 between tumor and non-tumor samples. All data collection processes are shown in Fig. 2. Among the 9 microarrays GSE11416-GPL6244, GSE32395-GPL6244 and GSE68591-GPL11028, GSE69524-GPL110284, two microarrays were measured via the same platform, so they were integrated by R package sva. Box plots and ROC curves were drawn to illustrate the expression trend of CKS2 in OS and its diagnostic ability of identifying OS patients acted as a proto-oncogene (Additional file 1: Figs. S1, S2). The SMD and sROC curves of the combined microarray data indicated the overexpression of CKS2 in OS samples (Additional file 1: Fig. S3A, SMD = 1.38, 95%CI [− 0.01–2.77]), and the significance of CKS2 expression making a distinction between OS and non-tumor samples (Additional file 1: Fig. S4A, Sensitivity = 0.92[0.59–0.99], Specificity = 0.88[0.49–0.98], Additional file 1: Fig. S4B, AUC = 0.96 95%CI [0.93–0.97]), which was consistent with the IHC. The Begg’s test showed no obvious publication bias (*P* = 0.174, Additional file 1: Fig. S3B). Meanwhile, the overexpression and discrimination ability of CKS2 in OS were further confirmed via comprehensive curves of SMD and sROC combined with IHC data (Fig. 3A, SMD = 1.57, 95%CI [0.27–2.86], Fig. 3B, AUC = 0.97, 95%CI [0.95–0.98]).

**Table 1:** Basic information for all included OS datasets

Cohorts	Year	Country	Platform	OS sample	Non-tumor sample	Type
Microarray in-house	2019	China	Arraystar Human LncRNA Microarray v4.0	3	3	Tissue
TARGET-OS	2019	USA	Illumina	88	0	Tissue
E-MEXP-3628	2012	Israel	HG-U133	14	4	Tissue
GSE19276	2010	Australia	GPL6848	44	5	Tissue
GSE21257	2011	Norway	GPL10295	53	0	Tissue
GSE14827	2010	Japan	GPL570	27	0	Tissue
GSE16091	2009	USA	GPL96	34	0	Tissue
GSE39055	2013	USA	GPL14951	37	0	Tissue
GSE11416	2009	Canada	GPL6244	4	2	Cell line
GSE32395	2011	Germany	GPL6244	7	2	Cell line
GSE68591	2015	USA	GPL11028	10	2	Cell line
GSE69524	2015	USA	GPL11028	10	2	Cell line
GSE36004	2012	Norway	GPL6102	19	6	Cell line
GSE42352	2012	Norway	GPL10295	19	15	Cell line
GSE42572	2015	Norway	GPL13376	7	5	Cell line

### Association between up-regulation of CKS2 expression and clinical features of OS

**Fig. 4:**
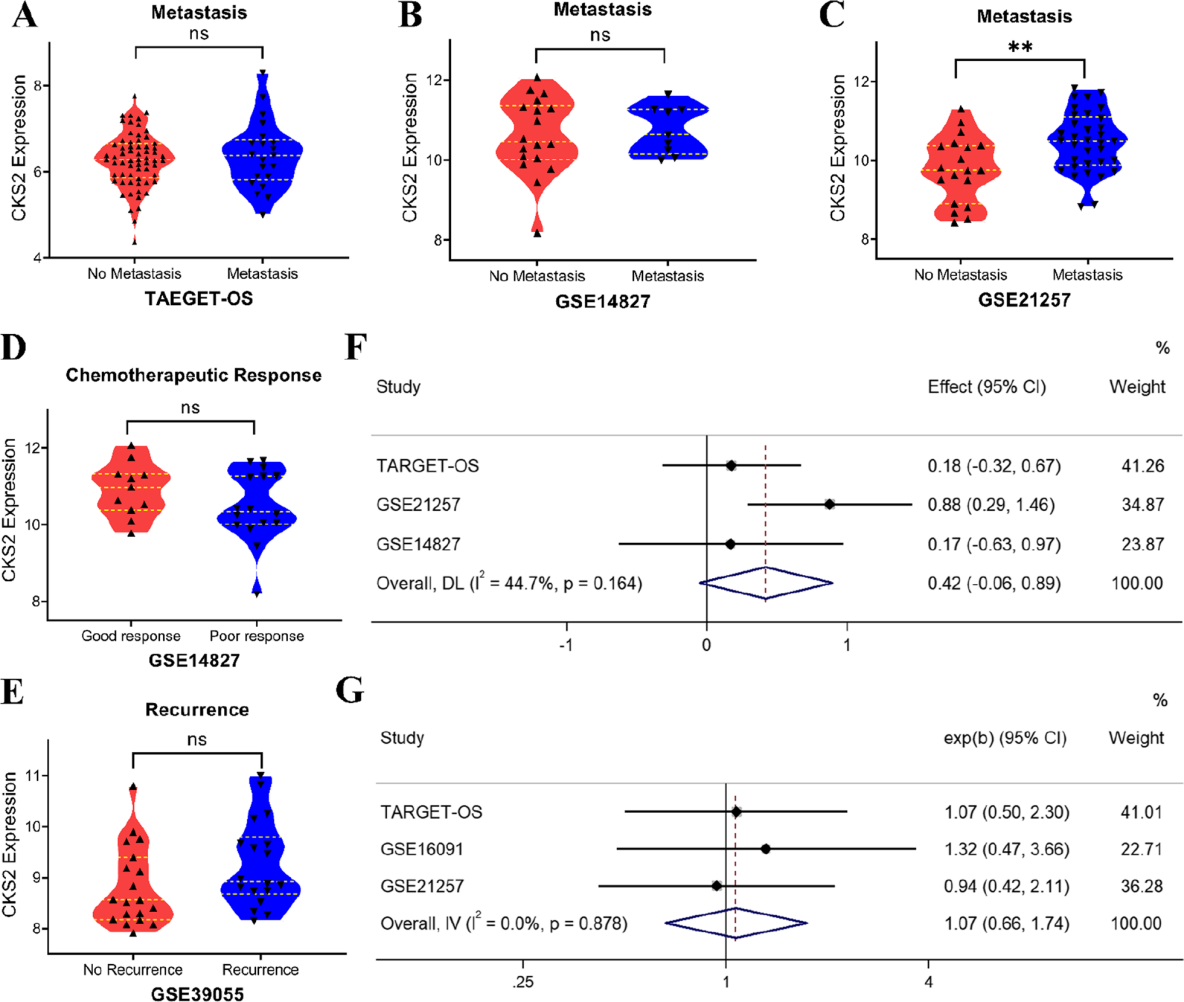
The clinic-pathological significance of CKS2 in osteosarcoma (OS). **A** Relationship between CKS2 expression and tumor metastasis in TARGET-OS. **B** Relationship between CKS2 expression and tumor metastasis in GSE14827. **C** Relationship between CKS2 expression and tumor metastasis in GSE21257. **D** Relationship between CKS2 expression and tumor recurrence in GSE39055. **E** Relationship between CKS2 expression and chemotherapeutic response in GSE14827. **F** Pooled SMD Forest Plot for OS patients with or without tumor metastasis. **G** Pooled HR Forest Plot for OS patients included in survival analysis. (“ns” means *P* > 0.05, “∗” means *P* < 0.05, “∗∗” means *P* < 0.01)

Further screening of the datasets was carried out to include the datasets that would facilitate the correlation study between CKS2 expression and clinicopathological. 4 datasets containing clinical parameters were selected for correlation analysis of CKS2 and chemotherapy efficacy, tumor recurrence and metastasis (Fig. 4A–E). In GSE21257, the expression of CKS2 mRNA in patients with metastatic OS was significantly higher than patients with non-metastasis (Fig. 4C). In addition, summarized difference of CKS2 expression was calculated to demonstrate that CKS2 expression was up-regulated in patients with metastatic OS with no statistical significance (SMD = 0.42, 95%CI [− 0.06–0.89], Fig. 4F). TARGET-OS, GSE16091 and GSE21257 containing survival information of patients were included in survival analysis (Table 2) and Kaplan–Meier survival curve of 85 OS patients in TARGET-OS showed that CKS2 expression had no significant effect on survival rate of OS patients (Additional file 1: Figs. S5A, B, S6a) as well as GSE21257. The combined HR of the three datasets was 1.07 (95%CI [0.66–1.74], Fig. 4G). The KM curve of 34 patients from GSE16091 (Additional file 1: Fig. S5C) suggested that the prognosis of OS patients with high CKS2 expression may be different from that with low expression in the first 3-year after diagnosis. Because of the small sample size, the result failed to achieve statistical significance (*P* = 0.068), and prediction value of CKS2 for OS patients need further attention.

**Table 2:** Clinical parameters of OS patients from 3 datasets included in survival analysis

Characteristic	TARGET-OS (n = 88)	GSE21257 (n = 53)	GSE16091 (n = 34)
*Vital status, n (%)*			
Alive	56(63.64)	30(56.60)	19(55.88)
Dead	29(32.95)	23(43.40)	15(44.12)
Unknow	3(3.41)	NA	NA
*Age, n (%)*			
≥ 18	19(21.60)	20(37.74)	NA
< 18	69(78.41)	33(62.26)	NA
*Gender, n (%)*			
Male	51(57.95)	34(64.15)	NA
Female	37(42.05)	19(35.85)	NA
*Metastasis, n (%)*			
Yes	22(25.00)	34(64.15)	NA
No	66(75.00)	19(35.85)	NA
*Histological, n(%)*			
Osteoblastic	NA	32(60.38)	NA
Other	NA	21(39.62)	NA

### scRNA-seq analysis suggests that CKS2 is up-regulated on proliferating osteoblastic OS cells in OS tissue

**Fig. 5:**
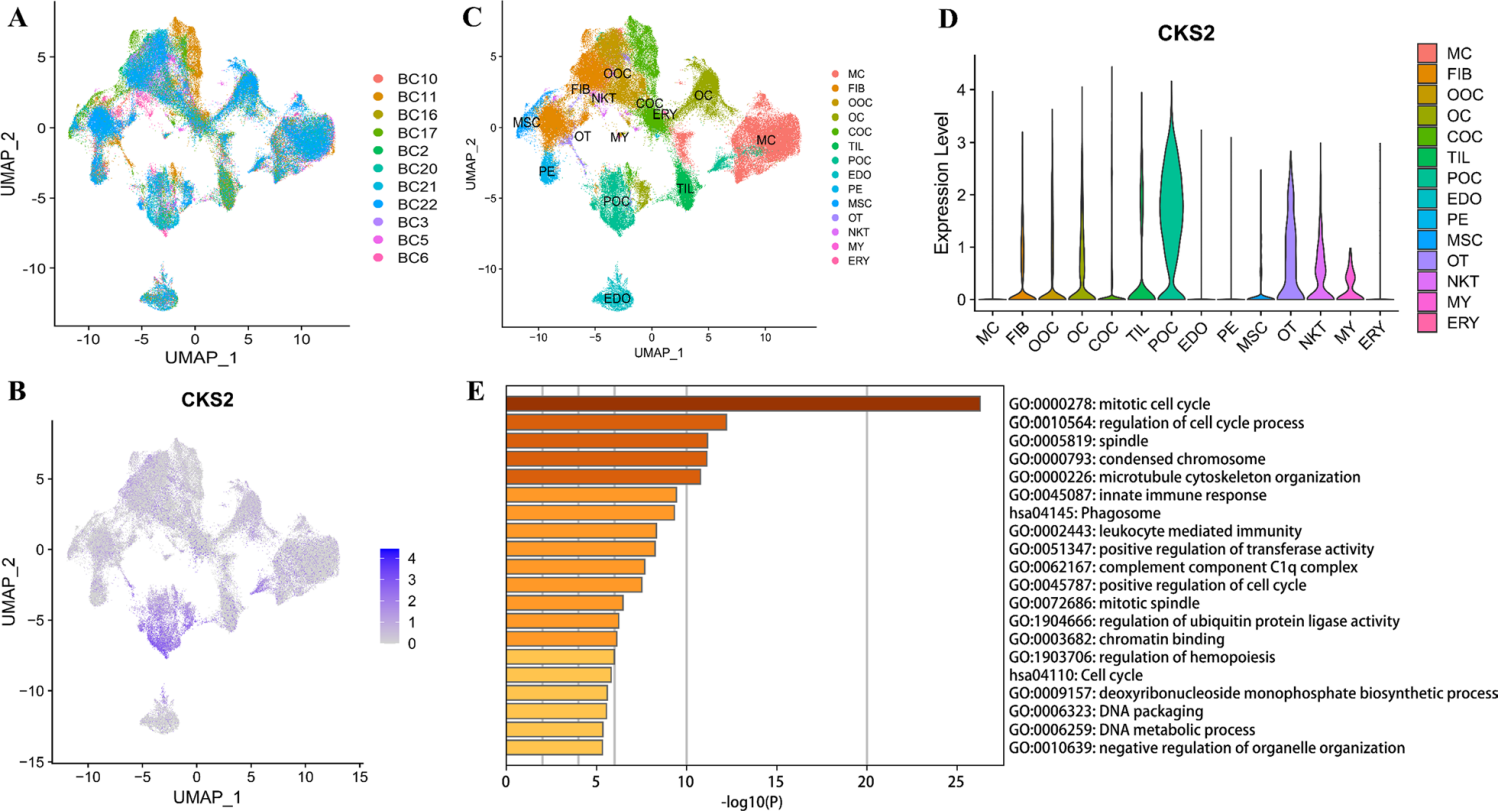
scRNA-seq analysis process of GSE152048. **A** Uniform manifold approximation and projection (UMAP) distribution of 11 samples. **B** Distribution of CKS2 expression in cell clusters. **C** UMAP distribution of annoted cell clusters through unsupervised clustering. **D** Violin-plot displaying distribution of CKS2 in various types of cells. **E** Function analysis of up-regulated genes on POC in OS tissues

The harmony function was adopted to rectify the sample heterogeneity of the expression matrix (Fig. 5A). The cells were identified as different clusters using an unsupervised clustering method, and the up-regulated molecules of the cell subpopulation were gained through differential screening. The up-regulated genes compared markers collected in the CellMarker database and marker molecules collected in the paper providing raw data. Overall, the cells subpopulation was marked as cells (Fig. 9A, D) in OS tissues (myeloid cells (MC), fibroblasts (FIB), osteoblastic OS cells (OOC), osteoclastic cells (OC), chondroblast OS cells (COC), tumor Infiltrating lymphocytes (TILs), proliferating osteoblastic OS cells (POC), endothelial cells (EDO), pericytes (PE), mesenchymal stem cells (MSC), Osteocyte (OT), Natural killer T cell (NKT), myoblasts (MY), erythrocyte (ERY), (Fig. 5C). CKS2 was found significantly over-expressed in POC (Fig. 5B–D). The enrichment analysis of up-regulated genes on POC suggest that POC is not only the cell subgroup whose cell cycle is positively regulated, but also related to the immune response of the organism (Fig. 5E).

**Fig. 6:**
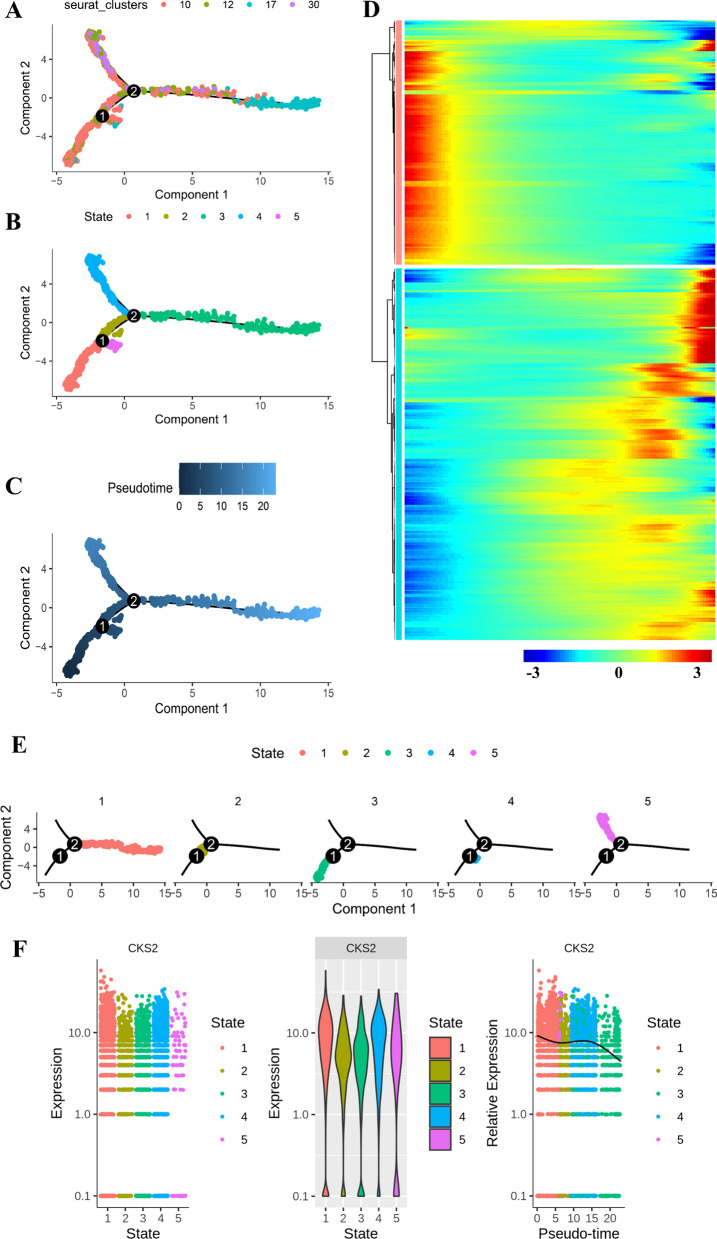
Trajectory analysis of POC in OS tissues. **A** The two-dimensional trajectory distribution map of cluster annotated on POC. **B** The two-dimensional distribution map of state shows the trajectory results of POC. **C** Display diagram of cell development timing score calculated according to development trajectory analysis of POC. **D** Expression pattern of genes defined as as influence factors of pseudo time and cluster = 2 (Red and cyan) was the best clustering effect parameter. **E** View the position of each state in the faceted track diagram. **F** The dynamic changes of the expression level of CKS2 during POC differentiation

Then, we selected genes with high degree of dispersion (mean_expression ≥ 0.1 and dispersion_empirical ≥ 1 * dispersion_fit) as order genes to define the development process of POC. Then the dimension of the data is reduced by using the reverse graph embedding (DDRtree) algorithm. Afterwards, trajectory construction was obtained according to the expression trend of order genes in way of three types, which were based on clusters gained by UMAP (Fig. 6A), different cell states (Fig. 6B), and pseudo time indicates the sequence of time (Fig. 6C). Then, differentialGeneTest function was used to find genes that change as influence factors of pseudo time. 435 genes were acquired (Related file 2) and num_clusters = 2 (Red and cyan) was the parameter to achieve the best clustering effect (Fig. 6D). The faceted trajectory diagram of cell state (Fig. 6E) and CK2 expression transforming with different cell states (Fig. 6F) showed a downward trend of CKS2 expression during the process of POC differentiation.

### CKS2 influenced vascular endothelial cells in TME

**Fig. 7:**
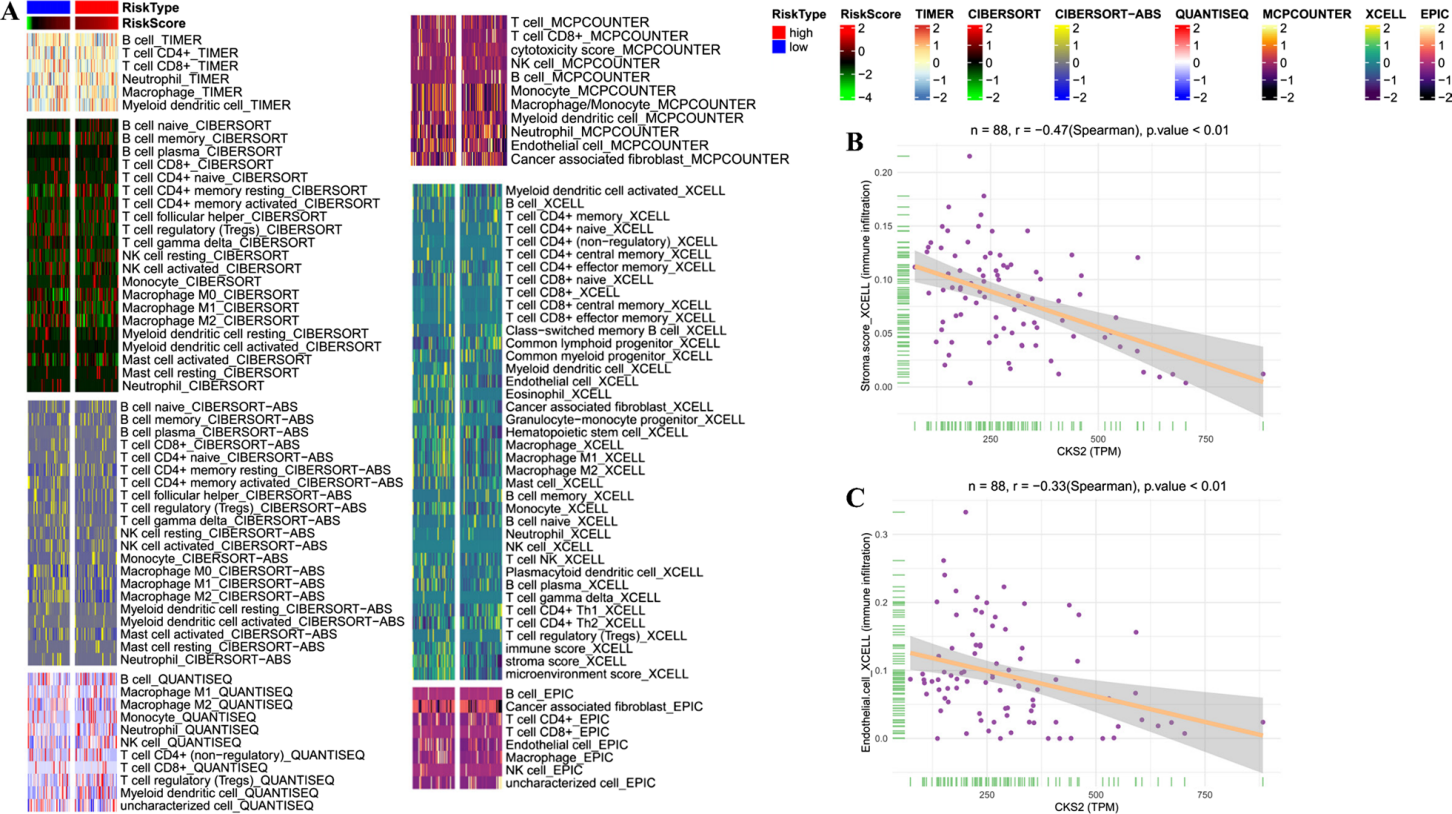
Results of immune infiltration analysis of patient from TARGET-OS. **A** Heatmap for immune responses based on CIBERSORT, ESTIMATE, MCPcounter, ssGSEA, and TIMER algorithms among patient of TARGET-OS. **B** Spearman correlation analysis showed that association between CKS2 and stroma score. **C** Spearman correlation analysis showed that association between CKS2 and Endothelial cell XCELL

Patients of TARGET-OS project was divided into two groups via median of CKS2 expression conducted as threshold. Based on algorithms including CIBERSORT, ESTIMATE, MCP counter and ssGSEA, the differences of immunoomics and TME was investigated between two groups (Fig. 7A). Because the predicted distribution of immune cells is non normal, Spearman correlation analysis was conducted rather than Pearson correlation analysis. The results showed that there was a remarkable negative correlation between CKS2 expression and stromal cell scores in TME as well as endothelial cell calculated by the Xcell algorithm (Fig. 7B, C). Simultaneously, stromal score and distribution of endothelial cell in patients with high-CKS2 were significantly lower than those in low-CKS2 group (Additional file 1: Fig. S6A, B).

### Co-expression Modular genes and enriched biological functions and pathways based on WGCNA

**Fig. 8:**
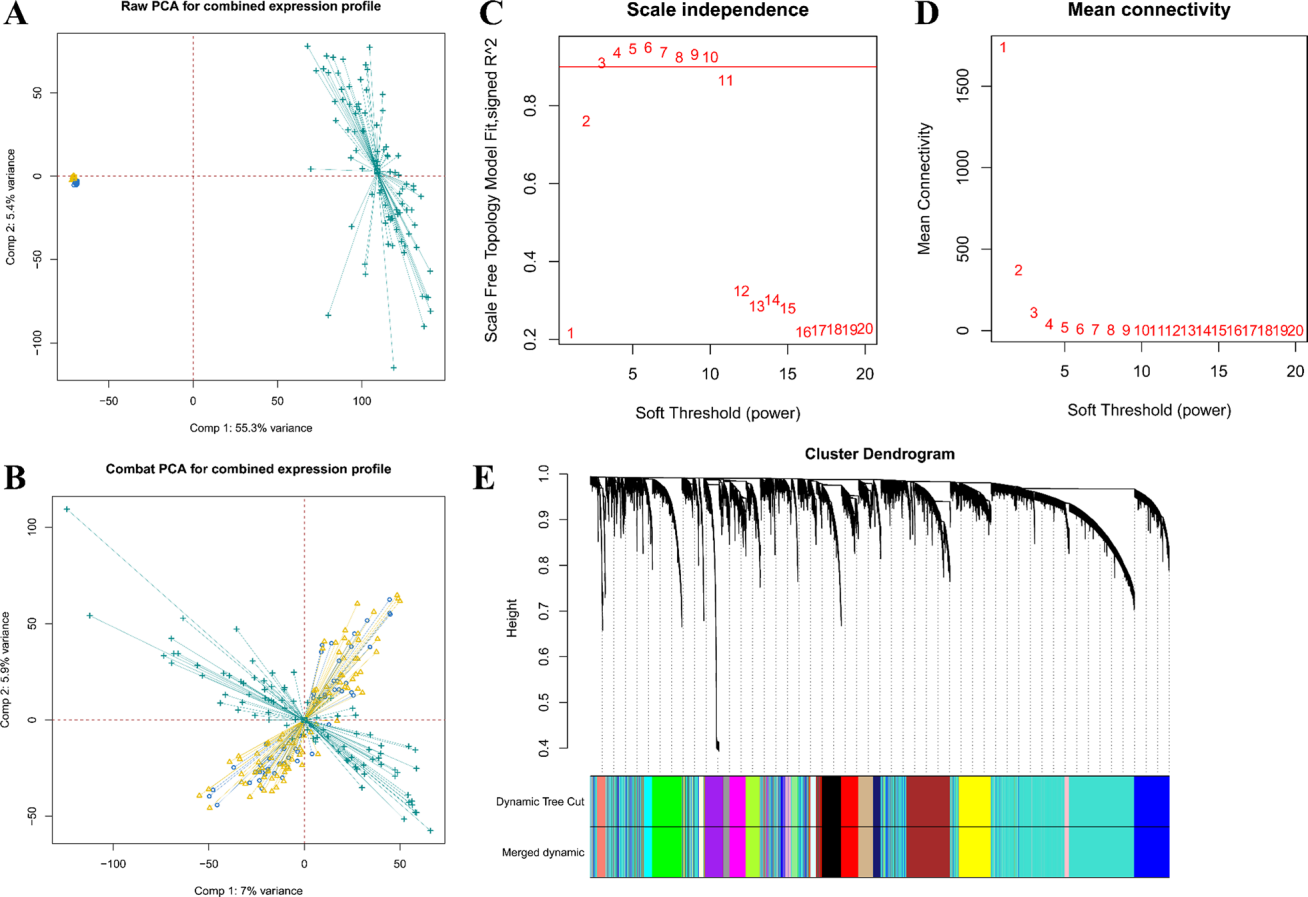
Weighted correlation network analysis (WGCNA) analysis was conducted to find co-expression genes of CKS2. **A**, **B** The “ComBat” algorithm was applied to reduce the likelihood of batch effects from non-biological technical biases between different datasets. **C** The function of soft threshold parameter and scale-free fitting index. **D** The function of soft threshold parameter and average connectivity. **E** Clustering system tree diagram of the combined gene matrix calculated by average hierarchical linkage clustering. The color squares below the tree diagram meant the modules cut by the dynamic tree

Through the ComBat function of the R package sva, TARGET-OS, GSE42352, and GSE21257 which datasets with large samples were integrated into a matrix of gene expression containing 225 patients with removed batch effect (Fig. 8A, B). The soft threshold was set to the smallest integer value when the fitting coefficient R^2^ reaches 0.9 meaning co-expression gene network attains the state of approximately scale-free network distribution. In this study, β = 3 was chosen as the soft threshold (Fig. 8C, D). Modules with highly similarity was combined via hcust function after Dynamic Tree Cut, and cluster dendrograms was generated (Fig. 8E). Based on the degree difference between genes, this study finally identified 21 independent modules. Among them, the module containing CKS2 had a total of 613 genes, which were included in the subsequent enrichment analysis and the top10 hub genes were listed in Additional file 1: Table S1.

The R package GOSemSim was applied to calculate the semantic similarity between GO terms, and enriched biological functions of co-expression genes were summarized as cell cycle, intercellular substance transport, innate immune response, ADP metabolic process, and planar cell polarity pathway (Additional file 1: Fig. S7).

**Fig. 9:**
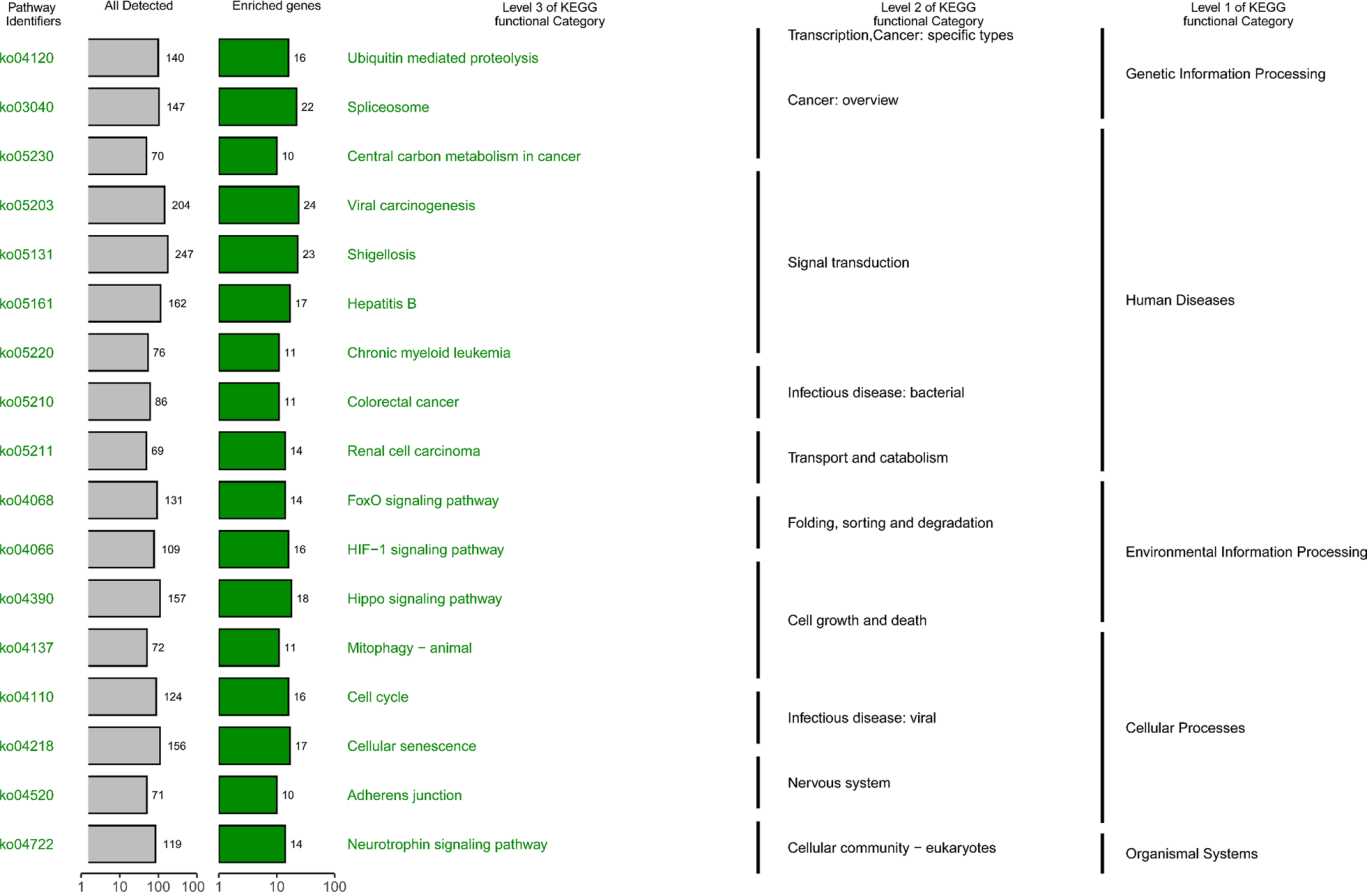
Kyoto encyclopedia of genes and genomes (KEGG) enrichment analysis for module genes co-expressed with CKS2, and KEGG terms are classified on 3 levels

Furthermore, in the light of pathway annotation from KEGG enrichment analysis, the enriched entries of co-expression genes were classified into cell function regulation, biological environment information process and genetic information process (Fig. 9).

## Discussion

OS is the most frequent bone tumor with high degree of malignancy. It originates from osteoblast mesenchymal cells, and regularly occurs in adolescents. According to the latest reports, the incidence of OS in Black, Hispanic and White adolescents was 6.8, 6.5, and 4.6/million, respectively [26]. Compared with other malignant tumors, the incidence of OS is relative low and the sample size of patients is scarce, simultaneously a considerable number of patients had not been diagnosed timely and effectively [27]. Therefore, the field has always been plagued for lack of large-scale studies on OS around the world. This study included 80 patients diagnosed OS, which provided strong support for this research field. At the same time, this study integrated data of microarrays and RNA-seq with IHC results to analyze the expression and clinicopathological significance of CKS2 in OS. What’s more, the research analyzed the correlations of CKS2 with various types of cells and component in TME of OS. In addition, the enriched biological functions of weighted co-expression genes of CKS2 were explored. Consequently, this study would offer significant insights into the clinical diagnosis and treatment of OS. At present, there was no research on the expression and clinical value of CKS2 in OS worldwide. Moreover, previous studies failed to comprehensively explore the clinical significance and molecular mechanism of CKS2 in OS, and analysis methods such as scRNA-seq, immune infiltration and WGCNA were scarcely employed to explore the potential biological mechanism of CKS2. This study filled the gaps through a systematic analysis based on support of multiple evidences.

We are the first team to compare the expression trend of CKS2 in OS and non-tumor tissues. Concurrently, some other prognostic markers in OS have been fully studied [28–30]. Although these studies have established reliable models related to the overall survival of OS patients through multiple machine learning methods, compared with our study, they lack the confirmation of candidate gene expression in OS and do not explore the gene expression on single-cell level. The evidence chain composed of our study materials includes analysis of differential expression profiling, SMD, and sROC containing IHC, microarrays, and public datasets showed that CKS2 is significantly up-regulated in OS and has a strong distinction ability between OS and non-tumor tissues. At the same time, datasets embodied the expression trend of CKS2 coming from six countries in four continents, suggesting that the abnormal up-regulation of CKS2 expression is common in organism, and it’s worthy of greater efforts for institutions worldwide to explore more intensive mechanism. Current studies found that OS patients suffering deferred diagnosis and treatment endured a higher risk of tumor metastasis and poor prognosis [31]. Therefore, including CKS2 in the diagnosis of OS will increase the chance of patients receiving effective diagnosis and treatment. Secondly, the high incidence of hematogenous metastasis and rapid progress of OS seriously threatened the lives of patients [32]. Therefore, it is urgent to find molecular markers that can reliably reflect the risk of metastasis in OS patients. Although the traditional high-throughput data showed that up-regulated CKS2 is inconsistent with the clinical characteristics of OS patients including metastasis, recurrence, chemotherapy response as well as survival, we found that CKS2 is highly expressed in POC through scRNA-seq analysis. Simultaneously, functional enrichment analysis suggests that the highly expressed genes in POC are enriched in the relevant biological process of cell cycle, suggesting that CKS2 promotes the metastasis of OS through positive regulation of cell cycle.

The existence of complex biological heterogeneity in OS results in poor sensitivity of OS patients receiving various therapies [33–35]. It had been confirmed that immune checkpoint treatment, such as inhibitors for Programmed Cell Death 1/CD274 Molecule, had limited effects on OS patients [36]. Therefore, it was urgent to seek for the causes for the dilemma and exploit new treatment strategy. TME is a special environment composed of extracellular matrix, immune infiltrating cells, stromal cells, blood vessels and lymphatic vessels, which is associated with the occurrence, development, and metastasis of tumor [37]. In this study, a variety of immune infiltration algorithms were applied to analyze the expression matrix in formation of TARGET-OS TPM. It was found that the expression of CKS2 was significantly negatively correlated with the stromal tissue score in the TME as well as the endothelial cells, and the interaction between vascular endothelial cells and tumor cells could often determine whether tumor cells were able to adhere to organs and tissues outside blood vessels and form the metastasis [38, 39]. Strilic et al. [40] found that tumor cells in humans and mice could induce programmed necrosis of endothelial cells, thereby promoting tumor cell extravasation and metastasis. The results of immune infiltration analysis in this study suggested that CKS2 is involved in the process of OS cells destroying the endothelial cell structure of the blood vessel wall, and caused metastases outside the blood vessel. In addition, the results of immune infiltration provided possible scientific hypotheses for the positive correlation between the up-regulation of CKS2 expression obtained in the previous steps and the development of OS. The complicated mechanism was worthy of further exploration.

At present, there was limited research on the regulatory mechanism of CKS2 in OS. Only Pan et al. [41] had analyzed the differentially expressed genes of 19 osteosarcoma cell lines and 4 non-tumor control tissue specimens through PPI network analysis and proposed that CKS2 might play an important regulatory role in OS. However, the study still had the following limitations: (1) The sample size was small, and OS cell line conducted as material was invalid to reflect the molecular pathological characteristics of OS compared with OS surgical specimens. (2) Although the construction of the PPI network is based on multiple methodology including experimental data, text mining of the literature database and the bioinformatics analysis [42], it failed to act OS as biological background. What’s more, PPI network analysis also has defects like insufficient analysis of the network's hierarchical structure and ignore of the relationship between functional modules and proteins [43, 44]. In this study, by eliminating the batch effect between different datasets, gene expression matrix containing 225 OS patients from TARGET-OS, GSE21257 and GSE42352 were processed by WGCNA to seek co-expression genes of CKS2. Meanwhile, the enrichment analysis of module revealed that the core genes including Dual Specificity Tyrosine Phosphorylation Regulated Kinase 1A (DYRK1A), Non-POU Domain Containing Octamer Binding (NONO), Thyroid Hormone Receptor Associated Protein 3 (THRAP3), Proteasome 26S Subunit, Non-ATPase 12 (PSMD12), etc. in co-expression module all have similar biological functions, which reflected the superiority of WGCNA for mining information. The enrichment analysis of this study showed that the genes of CKS2 co-expression module were significantly enriched in functions such as cell cycle, cell adhesion, and intercellular material transport, as well as biological pathways like Wnt signaling pathway and planar cell polarity pathway, which was consistent with the research of Rubin et al. [45] found that the abnormal activation of Wnt-pathway through OS cells caused the up-regulation of oncogene and tumor cell proliferation. Rubin went even further in his study to demonstrate that the intervention of Wnt inhibitory factor 1 (WIF-1) could significantly inhibit the metastasis of OS cells in mice. These findings suggested that interfering with the Wnt signaling pathway by targeting CKS2 might be one of the effective methods to suppress OS development.

Despite many encouraging findings were excavated in this study, current work still had some limitations. Our study emphasized the clinical significance of CKS2 in OS, but successive experiments in vitro and in vivo were still needed to further verify the biological role of CKS2 in OS and its upstream possible regulatory mechanism. In addition, although this study employed various methods to detect the expression of CKS2 in OS, status of CKS2 in the peripheral blood of OS patients had not been explored, thus whether the expression of CKS2 in peripheral blood had similar pattern to that in OS tissues and its clinical significance remained clarified. Concurrently, there were few studies exploring the diagnostic value of CKS2 in other tumors. Therefore, intensive evidence is required for clinical application of CKS2 via collecting serum or plasma samples from OS patients in subsequent studies.

## Conclusion

In summary, up-regulation of CKS2 expression in OS tissue was found through multiple technical approaches. In addition, scRNA-seq and co-expression analysis showed that CKS2 may have an impact on important biological process linked with cell cycle, cell adhesion, and intercellular material transport. Present study on CKS2 in OS indicated a promising prospect for CKS2 as a biomarker for OS.

## Supplementary Information

**Additional file 1.** Supplementary Figures S1-S7 and supplementary Table S1.

## Data Availability

The public data employed in the manuscript are stored in GDC (https://portal.gdc.cancer.gov/),GEO (https://www.ncbi.nim.nih.gov/geo/), and ArrayExpress (https://www.ebi.ac.uk/arrayexpress/).

## Abbreviations

OS: Osteosarcoma
CDK: Cyclin-dependent kinases
CKS2: Cyclin-dependent kinases regulatory subunit 2
pTNM: Pathological tumor-node metastasis
PPI: Protein–protein interaction
SC: Single-cell
IHC: Immunohistochemical
GDC: Genomic data commons
GEO: Gene expression omnibus
SMD: Standardized mean difference
ROC: Receiver operator characteristic
SROC: Summarized receiver operator characteristic
TPM: Transcripts per kilobase of exon model per million mapped reads
KM: Kaplan–Meier
HR: Hazard ratio
TME: Tumor microenvironment
PCA: Principal component analysis
UMAP: Uniform manifold approximation and projection
WGCNA: Weighted correlation network analysis
ME: Module eigengene
MM: Module membership
GO: Gene ontology
MF: Molecular function
BP: Biological process
CC: Cell composition
KEGG: Kyoto encyclopedia of genes and genomes
MC: Myeloid cells
FIB: Fibroblasts
OOC: Osteoblastic OS cells
OC: Osteoclastic cells
COC: Chondroblast OS cells
TILs: Tumor Infiltrating lymphocytes
POC: Proliferating osteoblastic OS cells
EDO: Endothelial cells
PE: Pericytes
MSC: Mesenchymal stem cells
OT: Osteocyte
NKT: Natural killer T cell
MY: Myoblasts
ERY: Erythrocyte
DRYK1A: Dual specificity tyrosine phosphorylation regulated kinase 1A
NONO: Non-POU domain containing octamer binding
THRAP3: Thyroid hormone receptor associated protein 3
PSMD12: Proteasome 26S subunit, Non-ATPase 12
WIF1: WNT inhibitory factor 1

## Declarations

### Related files

1. Clinical parameters of patient participating in the study. 2. 435 genes that change as influence factors of pseudo time in POC.
