# Supplementary material for: Clinicopathological value of the upregulation of cyclin-dependent kinases regulatory subunit 2 in osteosarcoma
Source: BMC Med Genomics. 2022 Apr 11;15:81. doi: 10.1186/s12920-022-01234-8 (PMC9004629; doi:10.1186/s12920-022-01234-8)
Supplement: Supplementary file 1 — Additional file 1. Supplementary Figures S1-S7 and supplementary Table S1. [file 12920_2022_1234_MOESM1_ESM.docx]

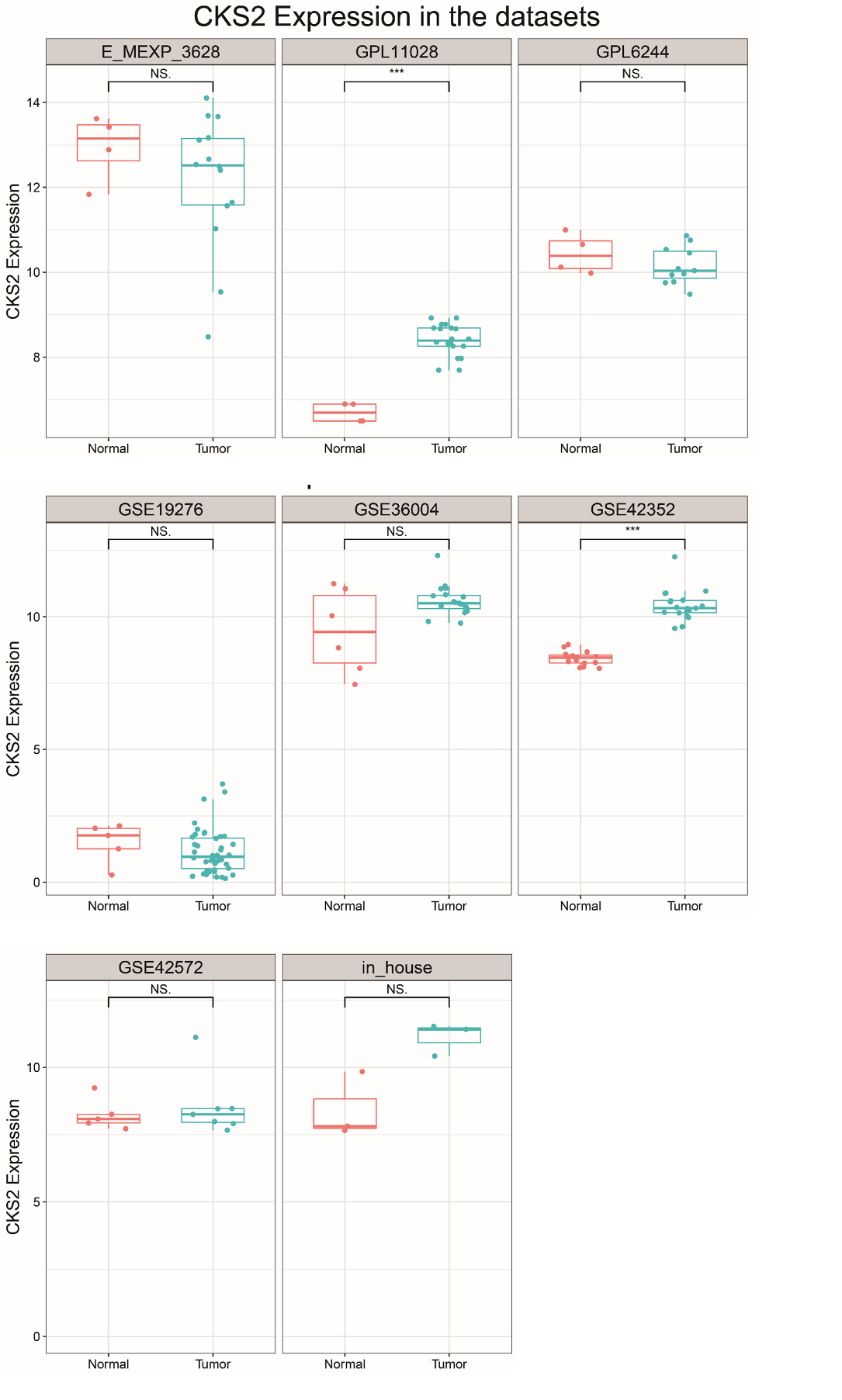


Figure S1: Box-plot displayed CKS2 expression in OS and non-tumor samples from in-house tissue microarrays and external microarrays. (N: Non-tumor, T: Tumor, “NS” means *P* >0.05, “∗∗∗” means *P* < 0.005).


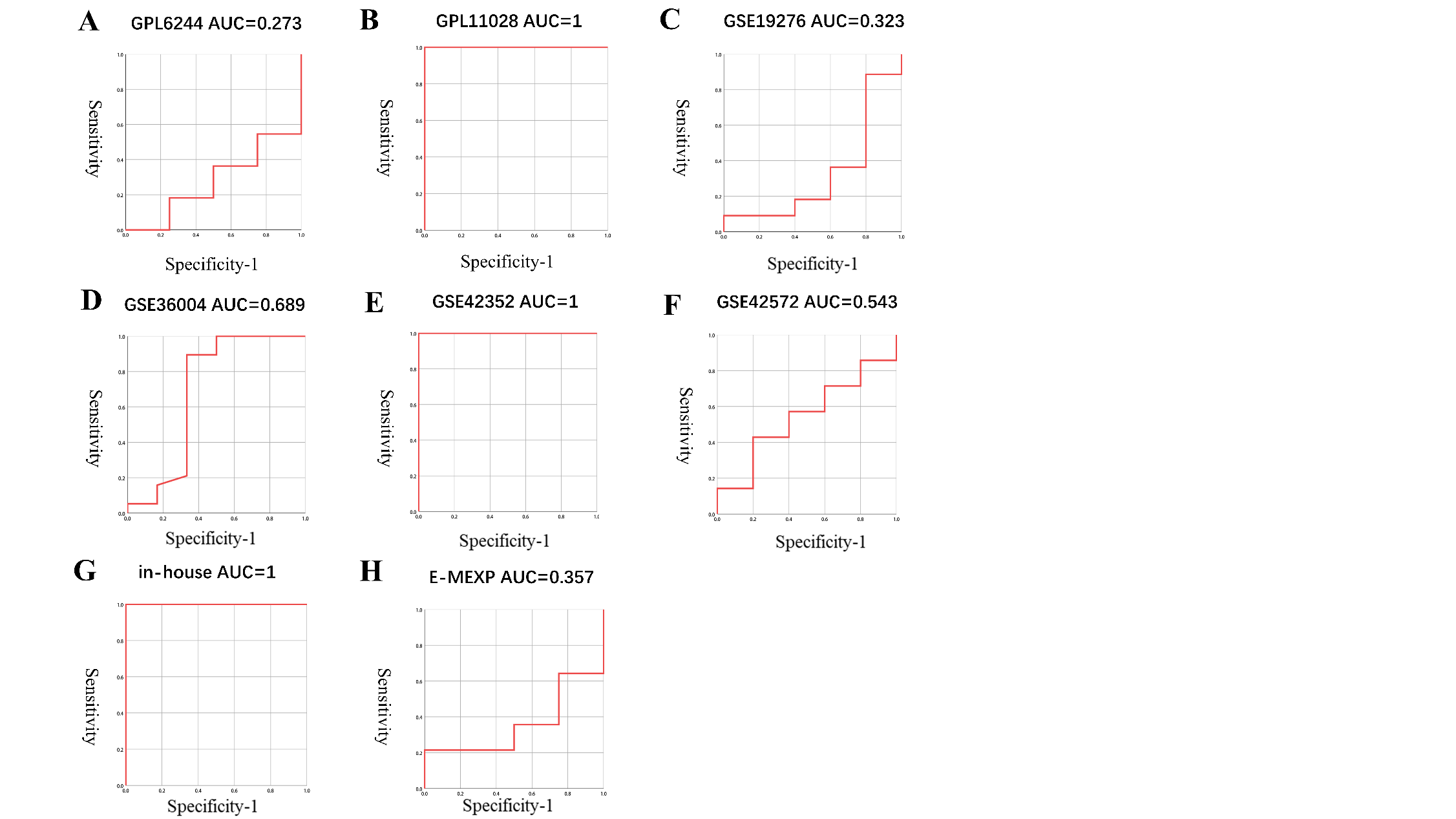


Figure S2: The ability of CKS2 expression in distinguishing OS from non-tumor tissues in each microarray dataset. (A)-(H) The receiver operator characteristic (ROC) curve of each dataset included in expression analysis of CKS2.


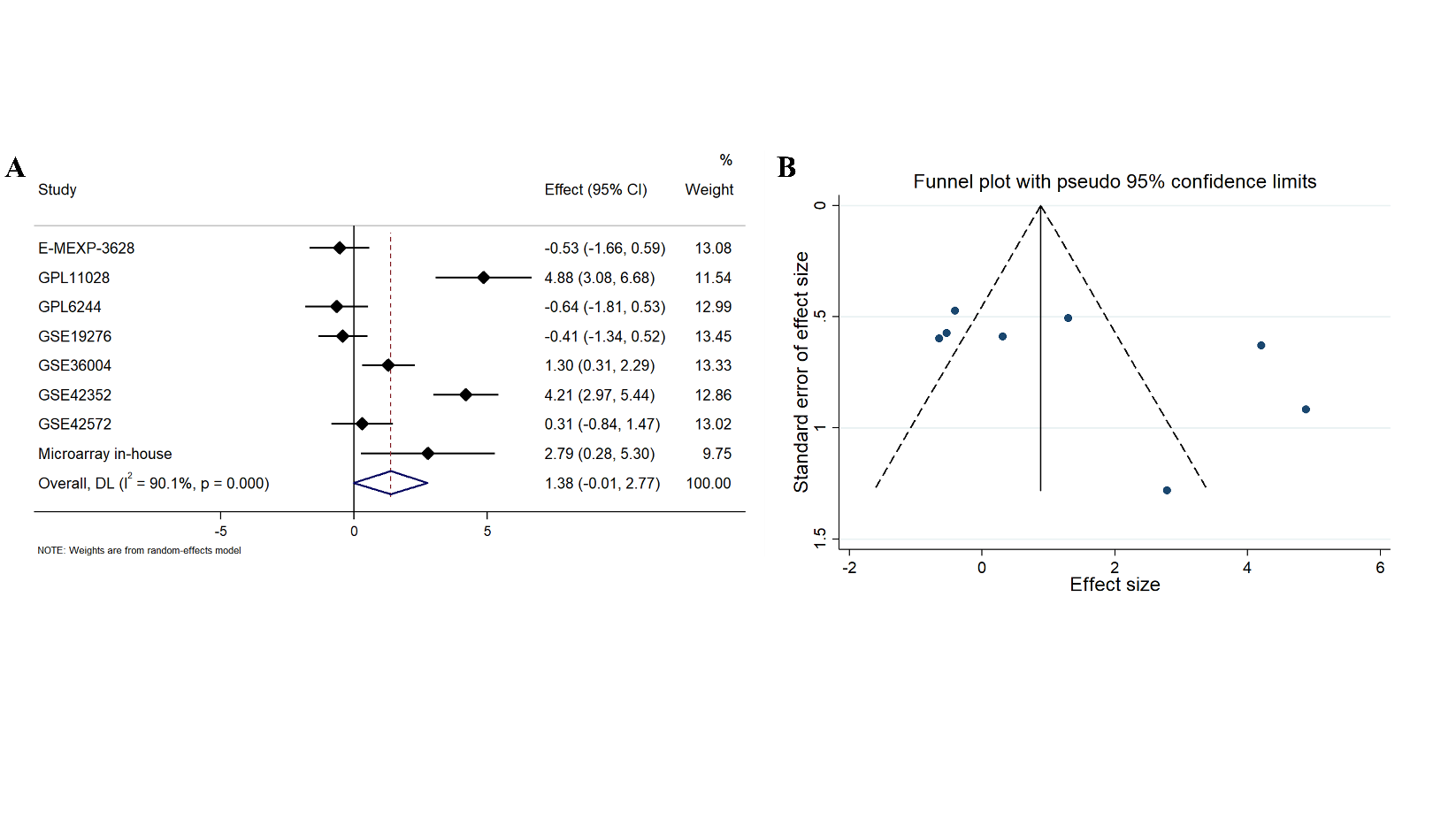


Figure S3: Integrative analysis of CKS2 expression in OS of each microarray dataset.

(A) Pooled SMD forest plot reflected overexpression of CKS2 in OS.

(B) Funnel chart of publication bias.


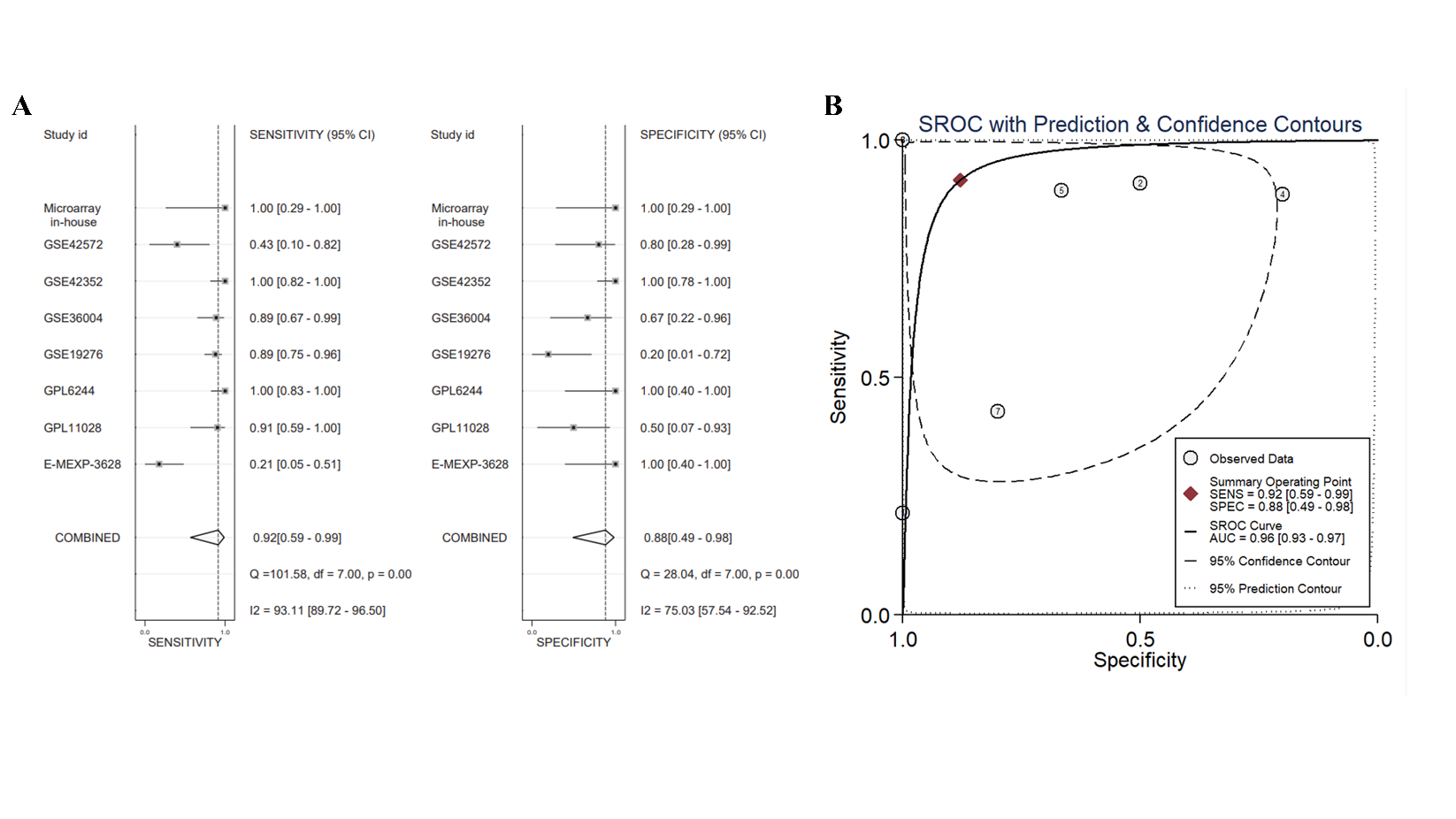


Figure S4: Diagnostic analysis of CKS2 expression in OS of each microarray dataset.

(A) Forest plot reflected the sensitivity and specificity of each microarray dataset.

(B) sROC curve reflected ability of CKS2 expression in distinguishing OS from non-tumor tissues.


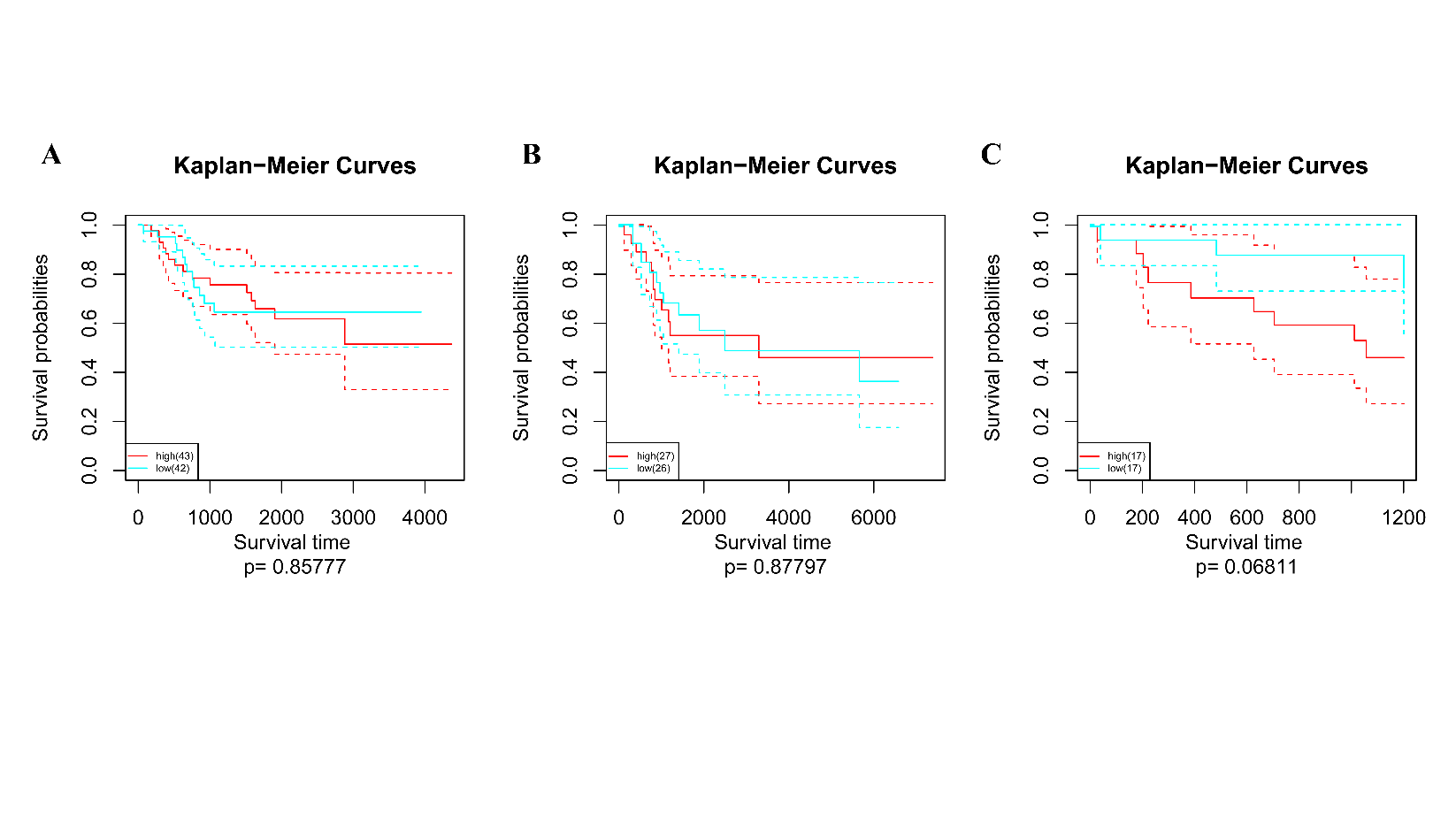


Figure S5: Survival analysis of CKS2 expression in OS patients from TARGET-OS, GSE21257 and GSE16091.

(A) Kaplan-Meier survival curves for overall survival of OS patients from TARGET-OS.

(B) Kaplan-Meier survival curves for overall survival of OS patients from GSE21257.

(C) Kaplan-Meier survival curves for overall survival of OS patients from GSE16091.


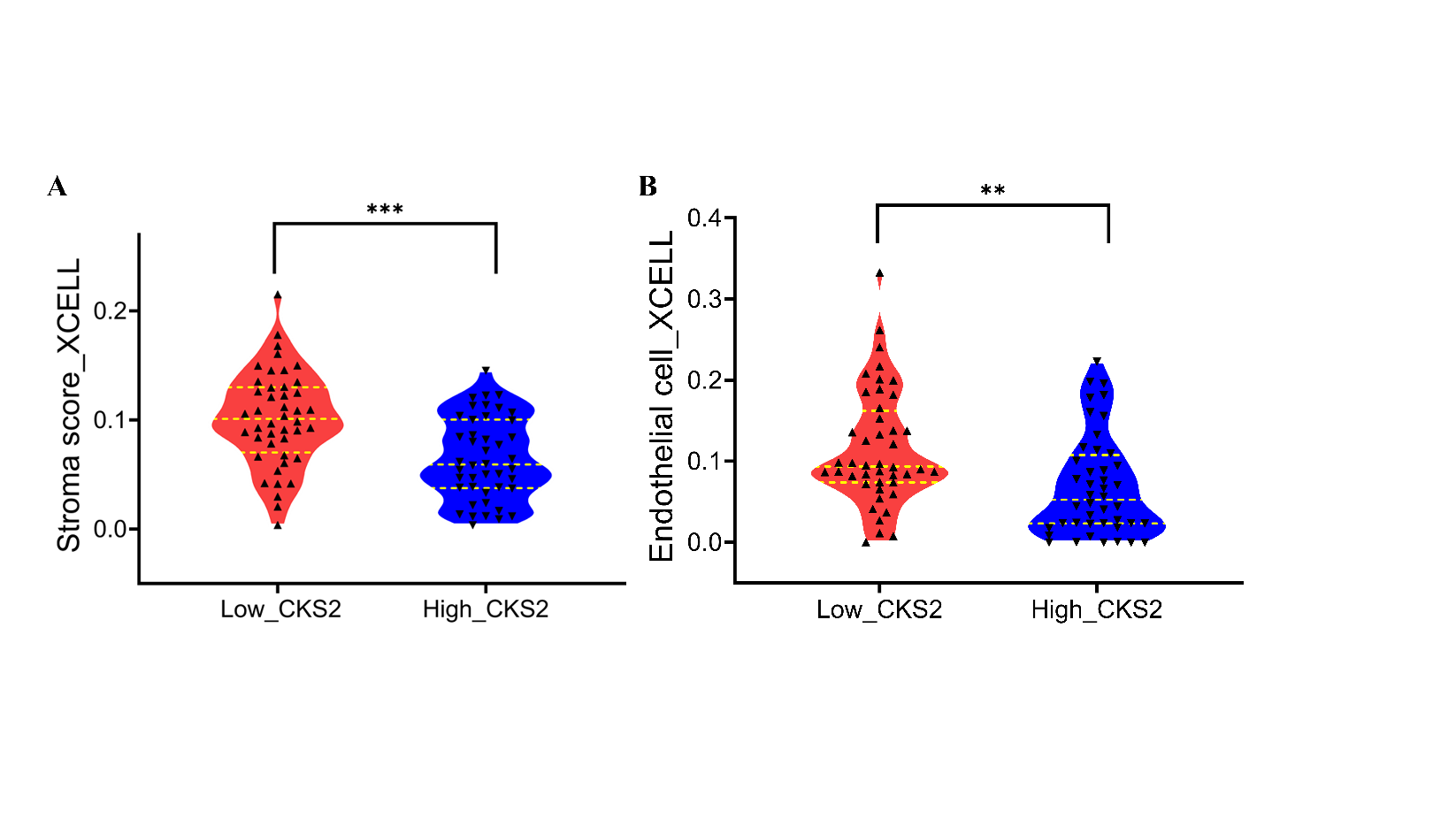


Figure S6: Violin plots for Immune infiltration analysis reflected distribution difference of stromal composition and immune cells between OS patient with low and high CKS2 expression.

(a) Violin plot for Stroma.score_XCELL in 2 subgroups.

(b) Violin plot for Endothelial.cell_XCELL in 2 subgroups.

(“∗∗” means *P* < 0.01, “∗∗∗” means *P* < 0.005)

TABLE S1: The top 10 hub genes selected according to the module membership analyzed by weighted correlation network analysis (WGCNA).

| Gene | Module Color | MM red | *P* -value of MMred |
| --- | --- | --- | --- |
| DYRK1A | red | 0.86 | 3.66E-47 |
| NONO | red | 0.86 | 2.61E-46 |
| THRAP3 | red | 0.85 | 6.28E-46 |
| PSMD12 | red | 0.85 | 2.25E-45 |
| PPIL4 | red | 0.85 | 9.99E-45 |
| TMPO | red | 0.85 | 1.02E-44 |
| ZNF146 | red | 0.84 | 4.16E-43 |
| SET | red | 0.83 | 7.37E-42 |
| ANP32E | red | 0.83 | 8.32E-42 |
| DDX6 | red | 0.83 | 3.35E-41 |

MM: Module membership.

**
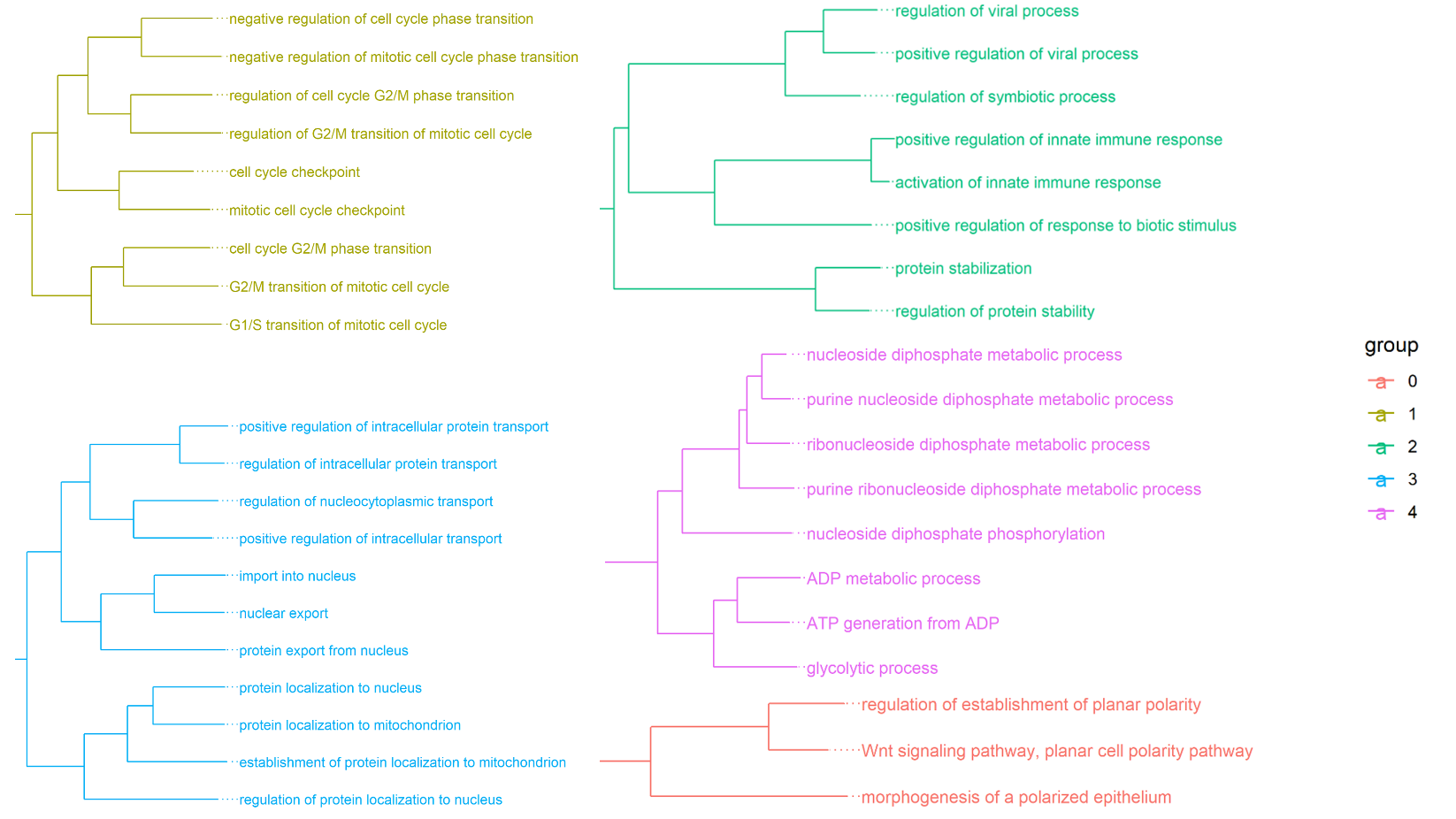
**

Figure S7: Via enrichment analysis of module genes, the enriched Gene Ontology (GO) terms are clustered into 5 classifications according to semantic similarity.
